# Protective Effects of Melatonin and Misoprostol against Experimentally Induced Increases in Intestinal Permeability in Rats

**DOI:** 10.3390/ijms23062912

**Published:** 2022-03-08

**Authors:** Karsten Peters, David Dahlgren, Péter Pál Egerszegi, Hans Lennernäs, Markus Sjöblom

**Affiliations:** 1 Department of Medical Cell Biology, Gastrointestinal Physiology, Uppsala University, 751 23 Uppsala, Sweden; karsten.peters@farmbio.uu.se (K.P.); david.dahlgren@farmbio.uu.se (D.D.); 2 Department of Pharmaceutical Biosciences, Translational Drug Discovery and Development, Uppsala University, 752 37 Uppsala, Sweden; hans.lennernas@farmbio.uu.se; 3 Department of Immunology, Genetics and Pathology, Clinical and Experimental Pathology, Clinical Pathology, Uppsala University, 751 85 Uppsala, Sweden; peter.pal.egerszegi@akademiska.se

**Keywords:** intestinal barrier dysfunction, single-pass intestinal perfusion, intestinal permeability, melatonin, misoprostol, gastrointestinal physiology

## Abstract

Intestinal mucosal barrier dysfunction caused by disease and/or chemotherapy lacks an effective treatment, which highlights a strong medical need. Our group has previously demonstrated the potential of melatonin and misoprostol to treat increases in intestinal mucosal permeability induced by 15-min luminal exposure to a surfactant, sodium dodecyl sulfate (SDS). However, it is not known which luminal melatonin and misoprostol concentrations are effective, and whether they are effective for a longer SDS exposure time. The objective of this single-pass intestinal perfusion study in rats was to investigate the concentration-dependent effect of melatonin and misoprostol on an increase in intestinal permeability induced by 60-min luminal SDS exposure. The cytoprotective effect was investigated by evaluating the intestinal clearance of ^51^Cr-labeled EDTA in response to luminal SDS as well as a histological evaluation of the exposed tissue. Melatonin at both 10 and 100 µM reduced SDS-induced increase in permeability by 50%. Misoprostol at 1 and 10 µM reduced the permeability by 50 and 75%, respectively. Combination of the two drugs at their respective highest concentrations had no additive protective effect. These in vivo results support further investigations of melatonin and misoprostol for oral treatments of a dysfunctional intestinal barrier.

## 1. Introduction

A healthy intestinal mucosa is a selective and dynamic barrier that separates the luminal contents from the systemic circulation [1]. This barrier consists of a mucus layer, a single layer of intestinal epithelial cells, and the underlying immune system. The role of the mucosa is to facilitate the transport of ions, nutrients, and water while preventing the infiltration and uptake of potentially harmful substances such as allergens, microbiota, and toxins. The most prevalent subgroup of cells in the intestinal epithelium are enterocytes, making up about 94% of the jejunal cell population [2]. They are shed and completely replaced every three to five days without any loss of barrier function [3]. The enterocytes maintain physiological functions in digestion and absorption by the means of carrier-mediated electrolyte and nutrient absorption and secretion. In addition, tight junction proteins at the luminal side of the epithelium regulate paracellular flux of hydrophilic molecules and electrolytes with low cell membrane permeability, in order to maintain intestinal and systemic homeostasis [4]. This is achieved through constant adaptation following hormonal, neural, and luminal stimuli, facilitated by the tight junction protein links to the intracellular actin cytoskeleton [5,6].

An inapt intestinal barrier shows, for instance, changes in epithelial absorptive and secretory functions as well as an increased mucosal permeability that can lead to the infiltration of harmful substances and bacteremia [7,8,9]. It is linked to a range of gastrointestinal (GI) and systemic conditions such as inflammatory bowel disease, Crohn’s disease, type 1 diabetes, and cholestatic liver disease as well as antineoplastic treatments with chemotherapeutics, tyrosine kinase inhibitors, and radiation [10]. This association highlights the importance of a viable and dynamic, healthy intestinal barrier. As there are no effective pharmacological treatments of an impinged epithelium available [10], there is a strong medical need to develop new therapies.

Melatonin released from the pineal gland has a significant physiological role in regulating the circadian rhythm. Interestingly, the greatest source of melatonin is the GI tract, where it is synthesized by the enterochromaffin cells [11]. While its physiological role in the GI tract is poorly characterized, melatonin has been shown to be a mucosal protective substance. For instance, it reduces ethanol- and radiation-induced increases in intestinal permeability. For ethanol, the melatonin effect is primarily mediated by the activation of G-protein coupled melatonin receptors (MT_1_ and MT_2_), whereas for radiation, it is mainly antioxidative [12,13].

Prostaglandins, especially of the E-type, play an important role in mucosal homeostasis and inflammation. They have a broad range of short- and long-term effects in the intestine such as the regulation of mucus production, mucosal blood flow, and bicarbonate secretion [14]. Misoprostol is a synthetic E-type prostaglandin analogue that inhibits the production and release of pro-inflammatory cytokines such as interleukin 1, and thus has a cytoprotective effect in the intestine [15]. As such, it is used to treat GI ulcers and mucosal perturbation induced by nonsteroidal anti-inflammatory drugs [16].

Sodium dodecyl sulfate (SDS) is an anionic surfactant commonly used as a pharmaceutical excipient in many oral dosage forms [17]. At the high concentration of 5 mg/mL in the intestinal lumen, SDS has the potential to alter epithelial barrier integrity and it has been shown to increase the permeability of different compounds in both the absorptive and secretory directions [18,19]. Recent research from our group has shown that concentrations of 100 µM of melatonin and 10 µM of misoprostol mitigate SDS-induced changes to the mucosal barrier in rats [19]. These effects were observed after 15-min luminal SDS exposure. However, it is unclear whether the protective effects of melatonin and misoprostol also remain at longer SDS exposure times, where more severe mucosal injury is expected. There is also uncertainty regarding at what luminal concentrations these two substances are effective.

The primary objective of this rat single-pass intestinal perfusion (SPIP) study was to evaluate the potential of different concentrations of melatonin (10 and 100 µM) and misoprostol (1 and 10 µM) to protect the jejunum from increases in permeability induced by 60-min luminal SDS exposure. Alterations in jejunal epithelial permeability were investigated by continuously monitoring intestinal blood-to-lumen clearance of ^51^Cr-labeled ethylenediaminetetraacetate (^51^Cr-EDTA), a well-established marker for studies of mucosal barrier integrity [20]. The secondary objective was to evaluate the effect of luminal SDS exposure on mucosal morphology, with and without concurrent melatonin and misoprostol treatment.

## 2. Results

**Figure 1:**
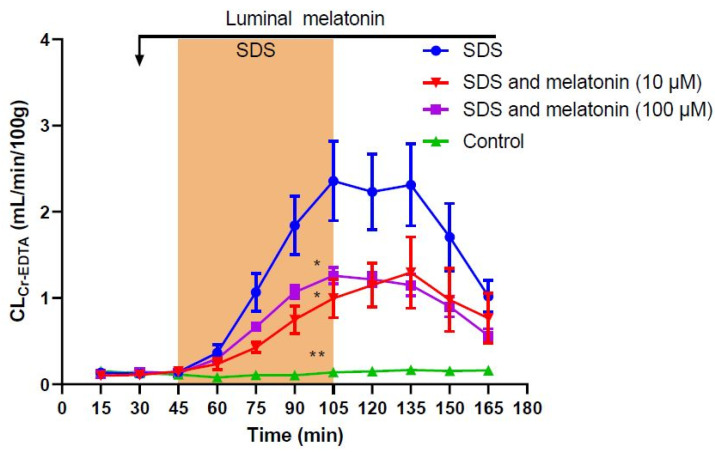
Effect of 5 mg/mL sodium dodecyl sulfate (SDS) in the luminal perfusate between 45 and 105 min on mean (±SEM) jejunal permeability (blood-to-lumen ^51^Cr-EDTA clearance (CL_Cr-EDTA_)). SDS induced a significant increase in permeability, an effect that was significantly reduced by the addition of luminal melatonin at concentrations of 10 µM and 100 µM. */** significantly (*p* < 0.05/*p* < 0.01) lower total CL_Cr-EDTA_ compared with the SDS group.

In the control group (i.e., animals perfused luminally with an isotonic (290 mOsm) phosphate-buffered solution (pH 6.5, 8 mM)), jejunal epithelial permeability of ^51^Cr-labeled ethylenediaminetetraacetate (^51^Cr-EDTA) was stable and low (0.08–0.16 mL/min/100 g) throughout the 165-min experiment. This resulted in a total CL_Cr-EDTA_ (between 45 and 165 min) of 14.0 ± 2.0 mL/100 g (Table 1, Figure 1). Perfusing the jejunal segment with an isotonic phosphate-buffered solution containing 5 mg/mL sodium dodecyl sulfate (SDS) for 60 min increased the total CL_Cr-EDTA_ about 15-fold to 204.0 ± 22.8 (*p* < 0.01, Table 1, Figure 1). The increase in CL_Cr-EDTA_ started directly when SDS was added to the perfusate at t = 45 min, and continued for 60 min until t = 105. When SDS was withdrawn at t = 105 min, the increase in CL_Cr-EDTA_ ceased and stayed at a plateau for 30 min. From there, it spontaneously recovered to reach 55% of the highest value at the end of the 60-min recovery period.

Use of luminal perfusate containing 100 µM melatonin significantly reduced (*p* < 0.05) the SDS-induced increase in total CL_Cr-EDTA_ by 50% when compared to SDS alone to 100.3 ± 5.7 mL/100 g (Table 1, Figure 1). The addition of melatonin at a lower concentration of 10 µM showed a similar effect and significantly reduced (*p* < 0.05) the SDS-induced increase in total CL_Cr-EDTA_ by about 50% when compared to SDS alone to 91.4 ± 18.2 mL/100 g (Table 1, Figure 1). There was no difference in effect observed between the two doses of melatonin (*p* > 0.05).

**Figure 2:**
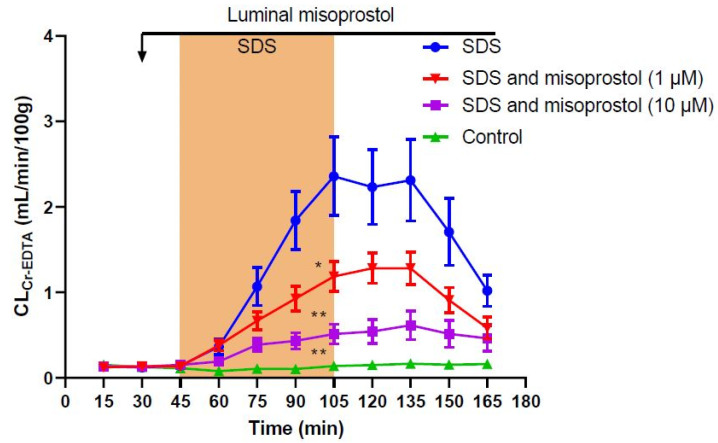
Effect of 5 mg/mL sodium dodecyl sulfate (SDS) in the luminal perfusate between 45 and 105 min on the mean (±SEM) jejunal permeability (blood-to-lumen ^51^Cr-EDTA clearance (CL_Cr-EDTA_)). SDS induced a significant increase in permeability, an effect that was significantly reduced by the addition of luminal misoprostol at concentrations of 1 µM and 10 µM. */** significantly (*p* < 0.05/*p* < 0.01) lower total CL_Cr-EDTA_ compared with the SDS group.

Addition of misoprostol at a concentration of 10 µM to the luminal perfusate significantly reduced (*p* < 0.01) the SDS-induced increase in total CL_Cr-EDTA_ by 75% compared to SDS alone to 50.0 ± 8.6 mL/100 g (Table 1, Figure 2). The addition of misoprostol at a lower concentration of 1 µM significantly reduced (*p* < 0.05) the SDS-induced increase in total CL_Cr-EDTA_ by 50% compared to SDS alone to 102.0 ± 10.6 mL/100 g (Table 1, Figure 2). A separate t-test showed a significant difference between the two misoprostol doses (*p* < 0.005).

**Figure 3:**
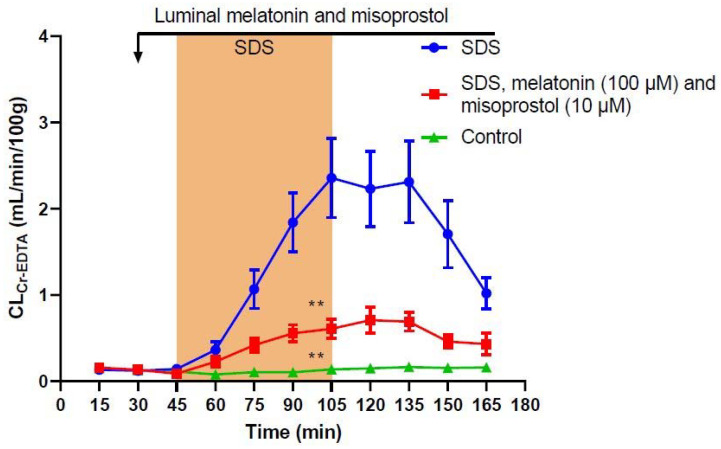
Effect of 5 mg/mL sodium dodecyl sulfate (SDS) in the luminal perfusate between 45 and 105 min on jejunal permeability (blood-to-lumen ^51^Cr-EDTA clearance (CL_Cr-EDTA_)). SDS induced a significant increase in permeability, an effect that was significantly reduced by the addition of a combination of luminal melatonin (100 µM) and misoprostol (10 µM). Values are means (±SEM). ** significantly (*p* < 0.01) lower total CL_Cr-EDTA_ compared with the SDS group.

To assess whether there would be an additive effect when combining both treatments, 100 µM melatonin and 10 µM misoprostol were perfused together. This significantly reduced (*p* < 0.01) the SDS-induced increase in total CL_Cr-EDTA_ by 75% when compared to SDS alone to 56.6 ± 7.3 mL/100 g (Table 1, Figure 3), exhibiting a similar effect as the higher misoprostol concentration (10 µM) alone.

**Figure 4:**
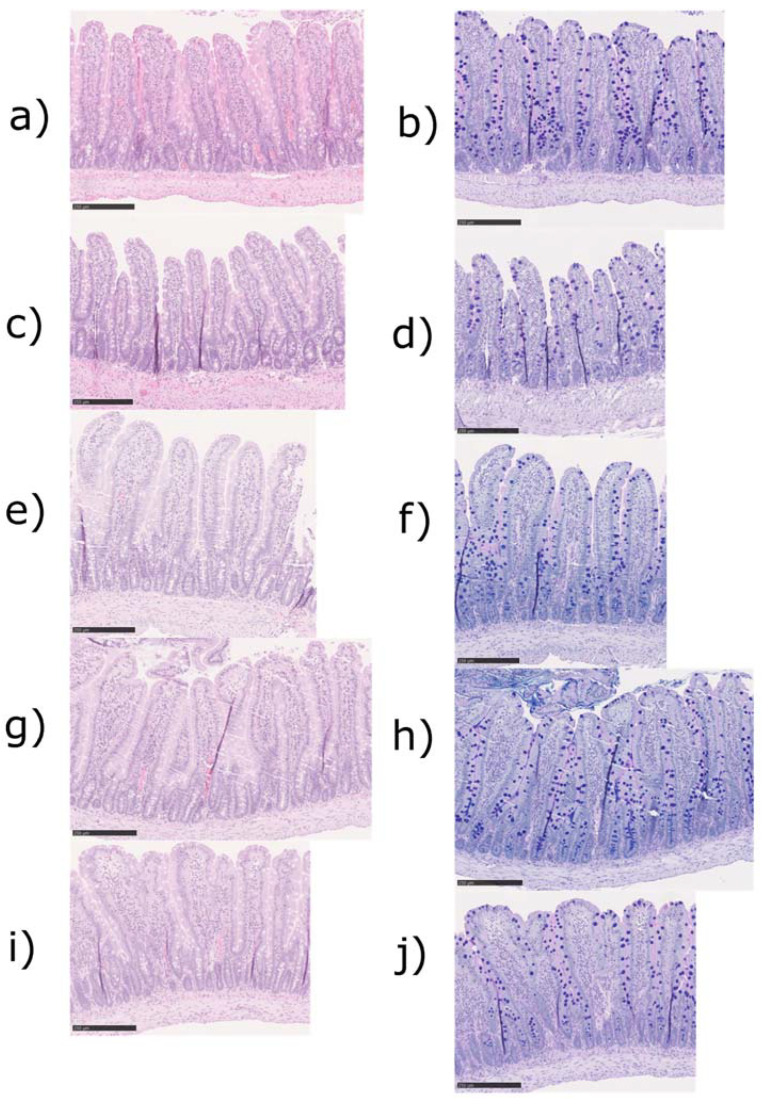
Histological images of the jejunal tissue from three different treatment groups with two different stainings, hematoxylin-eosin (**a**,**c**,**e**,**g**,**i**) and Alcian blue-PAS (**b**,**d**,**f**,**h**,**j**). Control animals only perfused with an isotonic phosphate-buffer solution (**a**,**b**). Animals perfused with sodium dodecyl sulfate (SDS) from 45 to 105 min of the experiment (**c**,**d**). Animals perfused with SDS from 45 to 105 min and melatonin (100 µM, **e**,**f**), misoprostol (10 µM, **g**,**h**) or a combination of the two (**i**,**j**) from 30 to 165 min of the experiment. Bars represent 250 µm.

Representative histological images of the jejunal tissues from the control, SDS alone, and SDS, melatonin, and misoprostol groups with two different stainings, hematoxylin-eosin (a,c,e) and Alcian blue-PAS (b,d,f) are displayed in Figure 4. The histological evaluation of the tissues gathered after the experiments (t = 165 min, Table 2) did not reveal any significant differences between any of the groups in histological injury parameters. The majority of animals displayed villi that were normal in length and width, light edema, no inflammation, a normal distribution of mucus, and no signs of apoptosis.

## 3. Discussion

This experimental in vivo study investigated the potential of two substances, melatonin and misoprostol, to treat a disrupted intestinal mucosal barrier induced by 60-min luminal exposure of the surfactant sodium dodecyl sulfate (SDS), in a single-pass intestinal perfusion (SPIP) model in rats [19]. The substance effects were investigated by continuously monitoring the blood-to-lumen clearance of ^51^Cr-labeled ethylenediaminetetraacetate (^51^Cr-EDTA) over time as well as evaluating the histology of the perfused intestinal segments.

Melatonin synthesized in the pineal gland is primarily known for its role in the circadian rhythm, but it is also produced in the gastrointestinal (GI) tract [11]. There, it is involved in, for instance, duodenal mucosal bicarbonate secretion, acid secretion, and regulation of basal duodenal and jejunal permeability [12,21]. While local physiological concentrations of melatonin are currently unknown and can only be estimated, the content in rat jejunal tissue was found to be approximately 0.5 ng/g [22]. Furthermore, release of melatonin in the duodenal segment induced by intracerebroventricular injection of phenylephrine generated an increase in luminal concentration of melatonin from 5 to 60 ng/mL (22 to 261 nM) [23], whereas the concentrations used in this study were about 2.3 and 23.2 µg/mL (10 and 100 µM). This is because the use of melatonin in this study was not meant to represent the effects of endogenous, but rather exogenous treatment. As a pharmaceutical, melatonin has been shown to have positive effects as a treatment of irritable bowel syndrome [24] and inflammatory bowel disease, in both mice [25] and humans [26]. In addition, it reduces increases in intestinal mucosal permeability induced by luminal exposure to ethanol and SDS [12,19]. These effects are mediated through agonistic activation of two G-protein coupled melatonin receptors (MT_1_ and MT_2_) [27], and can thus be abolished by the addition of the melatonin receptor antagonist luzindole [21,28]. However, other mechanisms of action have also been proposed. For instance, melatonin has been shown to be a potent scavenger of free radicals, and thus has protective effects on radiation-induced increases in intestinal permeability [13,29,30,31]. This process occurs both through direct detoxification of reactive oxygen and nitrogen species as well as by stimulating antioxidant enzymes and suppressing pro-oxidant enzymes [32]. Furthermore, in Caco-2 cells, melatonin attenuates IL-1β [33] or lipopolysaccharide [34] induced inflammation, characterized by increased levels of IL-6, IL-8, COX-2, and NO. It was suggested that the protective effect shown in the IL-1β-induction study was associated with a reduction in NFκB activation and that the reduced release of IL-6 was not melatonin-receptor-mediated as it could be abolished by luzindole addition. However, while proposing an antioxidative component to this effect, Mannino et al. (2019) allude to the need to further investigate the mechanisms [33].

In this 60-min SDS exposure study, there was no clear dose response of melatonin, suggesting a maximum effect was already reached at 10 µM. Furthermore, the 50% reduction induced by the two melatonin concentrations (10 and 100 µM) was slightly lower than the reported 75% observed with 100 µM melatonin in response to a shorter 15-min SDS exposure [28]. This was to be expected given the 4-fold higher increase in CL_Cr-EDTA_ induced by 60-min compared to 15-min SDS exposure. Further investigations into effective doses and potential long-term treatment are needed to further elucidate these effects. Still, the ability of melatonin to also be effective at more extensive luminal exposure to SDS illustrates its remarkable capability to also alleviate GI injury at challenging conditions.

Misoprostol has been used for the prevention of nonsteroidal anti-inflammatory drug-induced ulcers and mucosal erosions [16,35]. It is a synthetic E-type prostaglandin analogue that unfolds its effects through the G-protein-coupled prostaglandin E receptors 1–4, where it acts as an agonist. These receptors protect against mucosal damage and are involved in epithelial homeostasis [36]. The cytoprotective effects of misoprostol are achieved through the regulation of gastric acid secretion, mucus secretion, and downregulated production of pro-inflammatory cytokines such as IL-1, IL-6, IL-8, and TNF. It also acts through the activation of adaptive cell survival pathways [15,37]. A previous study from our group showed that misoprostol (10 µM) reduced increases in CL_Cr-EDTA_ after 15-min jejunal exposure to SDS by 50% [19]. In the present 60-min SDS exposure study, misoprostol (10 µM) reduced the increase in CL_Cr-EDTA_ by 75%. This greater reduction after a longer exposure to SDS could be explained by the more substantial increase in CL_Cr-EDTA_. As a greater disruption occurred, misoprostol may have a higher potential in the reduction in pathological increases in intestinal permeability than could be shown in the 15-min setup. A lower dose of misoprostol (1 µM) had a lower, but still substantial effect, reducing the SDS-induced increase in CL_Cr-EDTA_ by 50%. This showed a clear dose response that had not been previously described within this setup. While the difference in magnitude of the effect warrants further investigation, misoprostol showed a notable potential for the treatment of pathologically increased intestinal permeability.

Based on the different mechanisms of action of melatonin and misoprostol, we decided to investigate their potential synergistic effect. A previous SPIP study showed an additive effect of melatonin (100 µM) and misoprostol (10 µM) [19] on an increase in CL_Cr-EDTA_ induced by 15-min SDS exposure. However, in this 60-min SDS exposure study, any potential additive effect was obscured as the drug combination provided the same effect as 10 µM misoprostol alone. Further experiments with a combination of lower doses of melatonin and misoprostol could elucidate the potential to use these drugs together to treat a dysfunctional intestinal mucosa. Additionally, experimental designs with a longer period would enable the regulatory effects of misoprostol as well as the antioxidant effect of melatonin to fully unfold, and should thus be further investigated.

Common observations associated with an injured or dysregulated intestinal barrier are an increased mucosal permeability, systemic infiltration of harmful substances, and potentially sepsis and multi-organ failure [7,8,38]. A disrupted mucosal barrier is thus identified as a potential therapeutic target [10]. In this study, we used the pharmaceutical surfactant SDS to induce partial intestinal barrier dysfunction, which has been reported to increase the GI absorption of low permeability drugs [17,39] in a concentration- and time-dependent manner [40,41]. It acts by incorporating into the lipid bilayer of the epithelial cells, which leads to destabilization of the cell membrane and the tight junction complex [39,42,43]. An oral bolus or intestinal perfusion in rats with 10 mg/mL SDS leads to villus shortening, erosion, and eruption [40,44]. However, in this SPIP study where the intestine was exposed to SDS at 5 mg/mL for 60 min, no effect was observed on the morphological parameters, in accordance with data following 15-min exposure [19]. This is consistent with reported data on an ethanol-induced increase in CL_Cr-EDTA_, where no morphological changes were observed during similar experimental conditions [12]. This indicates that substantial functional alterations to the intestinal mucosa occur in advance of macroscopic changes, which is of relevance for the study of barrier dysregulation in a range of systemic and GI conditions and diseases.

The SPIP model has been extensively used for investigations of intestinal physiology, pathophysiology, and drug pharmacokinetics [45,46,47]. It has the advantage that it provides an intact intestinal blood supply and morphology as well as neuroendocrine and hormonal signaling. These are all important components in both the development and treatment of mucosal barrier dysfunction. However, laparotomic surgery performed in the SPIP model preparation causes postoperative ileus, which influences normal physiological GI functions such as permeability, osmoregulation and motility [48,49]. In this study, we therefore pretreated all rats with a selective COX-2 inhibitor [50] as this restores the GI functions affected by surgery [49].

The remarkable ability of melatonin and misoprostol to affect the mucosal barrier illustrated in this study will be translated into clinically relevant conditions such as chemotherapy-induced mucositis [51]. The highly proliferating tissue and the rapid turnover of epithelial cells makes the intestinal mucosa especially vulnerable to chemotherapeutic treatment. In patients, this can lead to a large variety of pathologies, where some affect the quality of life such as diarrhea and nausea, while others are potentially fatal such as sepsis or multi organ failure [9,52,53]. Despite this, there are currently no available effective treatments for this GI condition.

In conclusion, the present study demonstrated that both melatonin and misoprostol are effective at protecting and restoring the jejunal mucosa from surfactant-induced increases in small intestinal permeability. However, the combination was not more effective than misoprostol given as a single agent. All together, these new results support further investigations of these substances for the treatment of conditions related to increased intestinal permeability such as chemotherapy-induced mucositis.

## 4. Materials and Methods

### 4.1. Chemicals and Drugs

Accustain^®^ formalin solution (10%, neutral buffered), ethanol, 5-ethyl-5-(1′-methyl-propyl)-2-thiobarbiturate (Inactin^®^), melatonin, and sodium dodecyl sulfate (SDS) were purchased from Sigma-Aldrich (St. Louis, MO, USA). Misoprostol was purchased from Tocris Bioscience (Bristol, UK). Sodium phosphate dibasic dihydrate (Na_2_HPO_4_∙2H_2_O), potassium dihydrogen phosphate (KH_2_PO_4_), sodium hydroxide (NaOH), and sodium chloride were purchased from Merck KGaA (Darmstadt, Germany). ^51^Cr-labeled ethylenediaminetetraacetate (^51^Cr-EDTA) was purchased from PerkinElmer Life Sciences (Boston, MA, USA). Parecoxib (Dynastat^®^) was obtained from Apoteket AB (Uppsala, Sweden).

### 4.2. Study Formulations

An isotonic (290 mOsm) phosphate-buffered (pH 6.5, 8 mM) perfusate solution was prepared with or without 5 mg/mL SDS (17.3 mM). Added to these two perfusate solutions were melatonin (10 or 100 µM), misoprostol (1 or 10 µM), or melatonin + misoprostol (100 µM + 10 µM). Ethanol stock solutions of melatonin (6.5 or 65 mM) and misoprostol (6.5 or 0.65 mM) were added to the perfusate solutions with final ethanol concentrations always below 0.5%. Osmolarity was determined by freezing-point decrement using a Micro Osmometer (Model 3MO; Advanced Instruments, Needham Heights, MA, USA).

Inactin^®^ was prepared at 500 mg/mL in deionized water. Parecoxib was prepared at 1 mg/mL in saline.

### 4.3. Animals, Anesthesia, and Surgery

The study was approved by the local ethics committee for animal research (no. C64/16) in Uppsala, Sweden. Male Han Wistar rats (strain 273) from Charles River Co. (Germany), body weight 295–530 g were used. Before the experiments, all animals were allowed to adapt to their housing for at least one week where they were allowed food and water ad libitum. Housing conditions were 21–22 °C at a 12 h–12 h light–dark cycle. Experimental setup and surgery have been previously described [54] but will be explained in brief. Rats were anesthetized with an intraperitoneal injection of Inactin^®^ (180 mg/kg). To reduce preoperative stress, anesthesia was performed by experienced staff at the animal department (Biomedical Center, Uppsala, Sweden), which had handled the animals previously.

Body temperature was kept stable at 37–38 °C throughout the experiments by a heating pad controlled by a rectal thermistor probe. An arterial catheter connected to a transducer-operated PowerLab system (AD Instruments, Hastings, UK) recorded systemic arterial blood pressure to control the general condition of the animals. An approximately 3-cm long abdominal incision was made and a jejunal segment (10–12 cm) was cannulated, placed outside of the abdomen [46], and covered with polyethylene wrap. To reverse surgery induced paralysis of the intestine, parecoxib 10 mg/kg was given intravenously (iv) after the surgery [50] as this is a prerequisite for investigating the effect of substances that act via the enteric nervous system.

### 4.4. Perfusion Study

After the surgery, ^51^Cr-EDTA was administered iv as a bolus of 75 µCi (0.4 mL), followed by a continuous iv infusion at a rate of 50 µCi per hour (1 mL/h) throughout the experiments. During the first 45 min after surgery, the jejunal segment was perfused with phosphate-buffered perfusate solution (pH 6.5, 8 mM, 37 °C) to allow for intestinal, respiratory, and cardiovascular functions to stabilize before initiating the experiments. The length and wet tissue weight of each intestinal segment were determined after each experiment. The luminal perfusion rate was at all times 0.2 mL/min (peristaltic pump, Gilson Minipuls 3, Le Bel, France).

**Figure 5:**
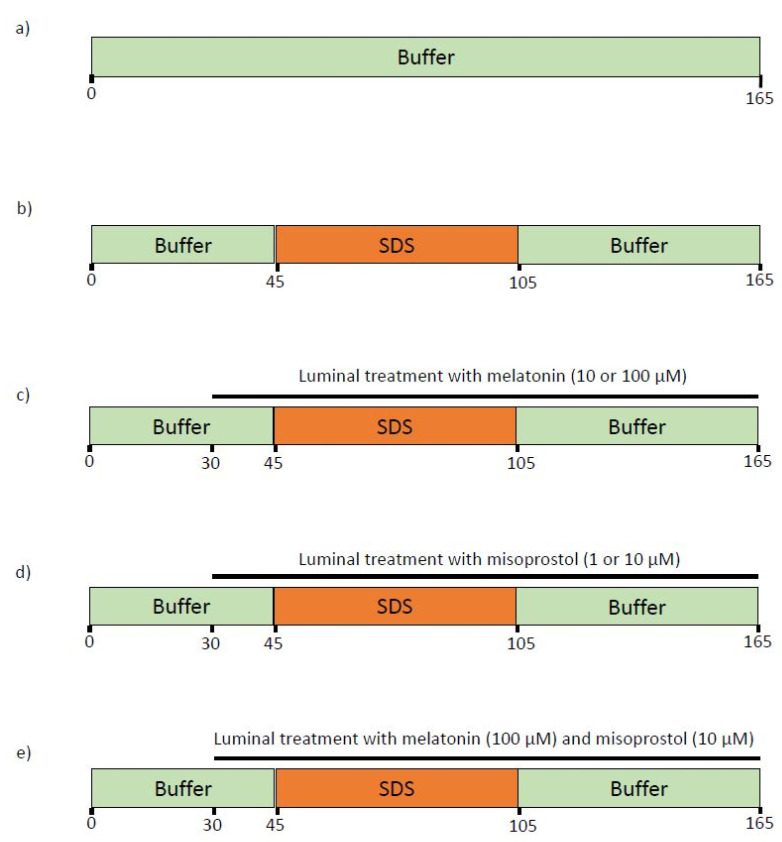
The luminal compositions, conditions, and treatments of the seven different experimental groups. The jejunal segment of rats (*n* = 6 in each group) was single-pass perfused with a pH 6.5 saline buffer solution without (**a**) or with (**b**–**e**) the addition of 5 mg/mL SDS between 45 and 105 min. Melatonin (10 or 100 µM, **c**), misoprostol (1 or 10 µM, **d**), or a combination of the two (100 and 10 µM, respectively, **e**), were supplemented from 30 min onward to investigate their effects on the increases in intestinal mucosal permeability induced by 60-min exposure of SDS.

After completion of the 45-min stabilization time, rats (*n* = 6) were assigned to one of the seven different experimental groups (Figure 5). In the control group, only the control buffer was perfused for 165 min. In the SDS group, the control buffer was perfused from 0–45 and from 105–165 min, while SDS was perfused from 45–105 min. In five SDS and luminal treatment groups, buffer and SDS were perfused in the same pattern as in the SDS group, and either melatonin, misoprostol, or melatonin and misoprostol were added to the perfusates from 30–165 min.

All experiments started with a quick filling (<30 s) of the entire perfused jejunal segment with the perfusate (37 °C, about 1.5 mL). The experimental setup was maintained at 37 °C. All exiting perfusate was collected and weighed at 15-min intervals. Blood samples (<0.3 mL) were taken from the femoral artery at the beginning (t = 0 min) and conclusion (t = 165 min) of the experiments. Blood samples were centrifuged (5000× *g*, 5 min) within 10 min, and the plasma and perfusates were analyzed for ^51^Cr activity.

### 4.5. Determination of Blood-to-Lumen Jejunal Mucosal ^51^Cr-EDTA Clearance

The luminal perfusates and blood plasma (at t = 0 and t = 165) collected during the experiments were analyzed for ^51^Cr-EDTA activity (cpm) in a gamma counter (1282 Compugamma, CS, Pharmacia AB, Sweden). To calculate corresponding plasma values for each time point, a perfusate sample was taken, and a linear regression analysis of the plasma samples was made.

The blood-to-lumen CL_Cr-EDTA_ was calculated using Equation (1) [55]:

(1) $${\mathrm{CL}}_{\mathrm{Cr}-\mathrm{EDTA}}=\frac{C_{\mathrm{perfusate}}\times {\;Q}_{\mathrm{in}}}{C_{\mathrm{plasma}}\times \mathrm{tissue}\;\mathrm{weight}}\times 100$$

where C_perfusate_ and C_plasma_ are the activities (cpm/mL) in the perfusate and plasma, respectively, and Q_in_ is the flow rate (mL/min) into the segment. CL_Cr-EDTA_ is expressed as mL/min/100 g wet tissue weight. For the evaluation of CL_Cr-EDTA_ over time, CL_Cr-EDTA_ values were normalized against the average value of all groups during the 45-min control period. To achieve a better comparison between groups during SDS exposure and the following recovery period, the area under the CL_Cr-EDTA_ over time curve between 45 and 165 min (total CL_Cr-EDTA_) was calculated using non-compartmental analysis in GraphPad Prism version 8.4.0 for windows (La Jolla, CA, USA).

### 4.6. Histology

For histological evaluation, samples were taken in five treatment groups, namely the control, SDS, melatonin (100 µM), and misoprostol (10 µM) as well as melatonin (100 µM) and misoprostol (10 µM). Three cross segments of the jejunum (one from the midpoint and two from regions close to either resection margin of the collected sample) were taken for evaluation. The sections were routinely prepared, cut into 3 µm slices, and stained with hematoxylin/eosin and Alcian blue-PAS (pH 2.5). An experienced gastrointestinal pathologist assessed the specimen in a blinded fashion using a light microscope. The following parameters were investigated: villus length and width; edema; mucus distribution; signs of inflammation; and apoptosis. All tissues were graded as: ( ) normal, (+) minor effect, and (++) major effect. The results from this evaluation were then condensed and are presented in Table 2. The grading was translated according to Table 3.

### 4.7. Statistical Analysis

A sample size of six rats was used in the CL_Cr-EDTA_ experiments based on previous studies. All descriptive statistics are presented as the mean ± standard error of the mean (SEM). The total CL_Cr-EDTA_ values of the different treatment groups were compared using a Brown–Forsythe and Welch ANOVA with a Dunnett multiple comparison test. In addition, the two doses of misoprostol were compared using a Welch’s t-test. Differences were considered statistically significant at a *p*-value < 0.05.

## Figures and Tables

**Table 1:** The mean (±SEM) area under the blood-to-lumen ^51^Cr-EDTA clearance (CL_Cr-EDTA_) time curve between 45 and 165 min (Total CL_Cr-EDTA_) of the seven different single-pass intestinal perfusion experiments. Two experimental substances—melatonin and misoprostol—were luminally administered, individually or in combination, to rats to evaluate their effects on sodium dodecyl sulfate (SDS)-induced increases in intestinal permeability. The *p*-values represent significant differences of the treatment groups from the SDS group.

Treatment	Total CL_Cr-EDTA_ (mL/100 g)	*p*-Value (Difference from SDS)
Control	14.0 ± 2.0	0.0050
SDS	204.0 ± 22.8	-
SDS + melatonin 10 µM	91.4 ± 18.2	0.025
SDS + melatonin 100 µM	100.3 ± 5.7	0.048
SDS + misoprostol 1 µM	102.0 ± 10.6	0.032
SDS + misoprostol 10 µM	50.0 ± 8.6	0.0068
SDS + melatonin 100 µM and misoprostol 10 µM	56.6 ± 7.3	0.0077

**Table 2:** Histological evaluation of the perfused jejunal tissues from five treatment groups. Sodium dodecyl sulfate (SDS), Not different from the control (Nd).

Treatment	Villi	Edema	Inflammation	Mucus	Apoptosis
Control	Normal	Minor effect	Normal	Normal	Normal
SDS	Nd	Nd	Nd	Nd	Nd
SDS + melatonin 100 µM	Nd	Nd	Nd	Nd	Nd
SDS + misoprostol 10 µM	Nd	Nd	Nd	Nd	Nd
SDS + melatonin 100 µM and misoprostol 10 µM	Nd	Nd	Nd	Nd	Nd

**Table 3:** Translation of blinded grading conducted by an experienced gastrointestinal pathologist.

Parameters	Normal	Minor Effect	Major Effect
Villus length and width	Normal height and width	Slightly shortened and widened	Strongly shortened and widened
Edema	No edema	Some small edema	Many and/or larger edema
Mucus distribution	Even distribution of mucus over the entire villi	Mucus lacking in small areas	Mucus lacking in large areas
Signs of inflammation	Absence of neutrophils	Infiltration of some neutrophils	Infiltration of a large number of neutrophils
Apoptosis	Normal number of apoptotic cells	Slightly increased number of apoptotic cells	Strongly increased number of apoptotic cells

## Data Availability

The data presented in this study are available on request from the corresponding author.
